# Clinical characteristics of 116 hospitalized patients with COVID-19 in Wuhan, China: a single-centered, retrospective, observational study

**DOI:** 10.1186/s12879-020-05452-2

**Published:** 2020-10-22

**Authors:** Shiqiang Xiong, Lin Liu, Feng Lin, Jinhu Shi, Lei Han, Huijian Liu, Lewei He, Qijun Jiang, Zeyang Wang, Wenbo Fu, Zhigang Li, Qing Lu, Zhinan Chen, Shifang Ding

**Affiliations:** 1 Department of Cardiology, the General Hospital of Central Theater Command, Wuhan, 430000 China; 2 Department of Integrated Traditional Chinese and Western Internal Medicine, the General Hospital of Central Theater Command, Wuhan, 430000 China; 3 Division of Medical Management, the General Hospital of Central Theater Command, Wuhan, 430000 China

**Keywords:** SARS-CoV-2, COVID-19, Cardiovascular disease, Hypertension, Coronary heart disease

## Abstract

**Background:**

A cluster of acute respiratory illness, now known as Corona Virus Disease 2019 (COVID-19) caused by 2019 novel coronavirus (SARS-CoV-2), has become a global pandemic. Aged population with cardiovascular diseases are more likely be to infected with SARS-CoV-2 and result in more severe outcomes and elevated case-fatality rate. Meanwhile, cardiovascular diseases have a high prevalence in the middle-aged and elderly population. However, despite of several researches in COVID-19, cardiovascular implications related to it still remains largely unclear. Therefore, a specific analysis in regard to cardiovascular implications of COVID-19 patients is in great need.

**Methods:**

In this single-centered, retrospective, observational study, 116 patients with laboratory-confirmed COVID-19 were enrolled, who admitted to the General Hospital of Central Theater Command (Wuhan, China) from January 20 to March 8, 2020. The demographic data, underlying comorbidities, clinical symptoms and signs, laboratory findings, chest computed tomography, treatment measures, and outcome data were collected from electronic medical records. Data were compared between non-severe and severe cases.

**Results:**

Of 116 hospitalized patients with COVID-19, the median age was 58.5 years (IQR, 47.0–69.0), and 36 (31.0%) were female. Hypertension (45 [38.8%]), diabetes (19 [16.4%]), and coronary heart disease (17 [14.7%]) were the most common coexisting conditions. Common symptoms included fever [99 (85.3%)], dry cough (61 [52.6%]), fatigue (60 [51.7%]), dyspnea (52 [44.8%]), anorexia (50 [43.1%]), and chest discomfort (50 [43.1%]). Local and/or bilateral patchy shadowing were the typical radiological findings on chest computed tomography. Lymphopenia (lymphocyte count, 1.0 × 10^9^/L [IQR, 0.7–1.3]) was observed in 66 patients (56.9%), and elevated lactate dehydrogenase (245.5 U/L [IQR, 194.3–319.8]) in 69 patients (59.5%). Hypokalemia occurred in 24 (20.7%) patients. Compared with non-severe cases, severe cases were older (64.0 years [IQR, 53.0–76.0] vs 56.0 years [IQR, 37.0–64.0]), more likely to have comorbidities (35 [63.6%] vs 24 [39.3%]), and more likely to develop acute cardiac injury (19 [34.5%] vs 4 [6.6%]), acute heart failure (18 [32.7%] vs 3 [4.9%]), and ARDS (20 [36.4%] vs 0 [0%]). During hospitalization, the prevalence of new onset hypertension was significantly higher in severe patients (55.2% vs 19.0%) than in non-severe ones.

**Conclusions:**

In this single-centered, retrospective, observational study, we found that the infection of SARS-CoV-2 was more likely to occur in middle and aged population with cardiovascular comorbidities. Cardiovascular complications, including new onset hypertension and heart injury were common in severe patients with COVID-19. More detailed researches in cardiovascular involvement in COVID-19 are urgently needed to further understand the disease.

## Background

In early December 2019, a cluster of acute respiratory illness, now known as Corona Virus Disease 2019 (COVID-19) caused by 2019 novel coronavirus (SARS-CoV-2), occurred in Wuhan, Hubei Province, China [1–4]. COVID-19 has rapidly spread all over the world and become a global pandemic. Although several recent studies have described the clinical spectrum of COVID-19, including general epidemiological characteristics, clinical manifestations, and clinical outcomes of patients [4–7], cardiovascular implications of COVID-19 remain largely unclear.

Cardiovascular diseases have a high prevalence in middle-aged and elderly population [8]. Aged population with comorbidities, such as cardiovascular diseases are more susceptible to COVID-19 and result in severe outcomes and elevated case-fatality rate [5, 9]. Acute cardiac injury is one of the common complications in COVID-19 patients [7]. These findings suggest that cardiovascular system is tightly implicated in COVID-19. By collecting data from 116 laboratory-confirmed cases who were admitted to the General Hospital of Central Theater Command, we sought to provide an up-to-date description of the clinical characteristics and cardiovascular status of patients with COVID-19. This study would not only identify the clinical manifestations with greater precision, but also provide assistance for non-cardiovascular specialists to manage COVID-19 patients with cardiovascular disorders.

## Methods

### Study design and participants

For this single-centered, retrospective, observational study, we recruited patients with laboratory-confirmed COVID-19 admitted to the General Hospital of Central Theater Command (Wuhan, China) from January 20 to March 8, 2020. A confirmed case with SARS-CoV-2 infection was defined as a positive result for real-time reverse-transcriptase polymerase-chain-reaction (RT-PCR) assay for pharyngeal swab specimens. The General Hospital of Central Theater Command is one of the major hospitals responsible for COVID-19 treatment designated by the government. At the time of admission, the severity of COVID-19 was defined as mild, moderate, severe and critical cases based on the 6th edition guideline issued by the National Health Commission of China. Mild patients were not admitted in this designated hospital. Moderate cases were having mild symptoms of respiratory infections with pneumonia. Severe cases were defined as dyspnea, respiratory frequency ≥ 30/min, blood oxygen saturation ≤ 93%, PaO2/FiO2 ratio ≤ 300 mmHg, and/or pulmonary inflammation progressing >50% within 24 to 48 h. Critical cases were those who exhibited respiratory failure, shock, and/or multiple organ dysfunction. In this study, moderate cases were categorized as the non-severe group; severe and critical cases were categorized as the severe group. Oral informed consent was obtained from patients. All the protocol in this study was approved by the Ethics Commission of the General Hospital of Central Theater Command ([2020]025–1). The clinical outcomes (ie, discharge, mortality, and length of stay) were monitored up to April 13, 2020, the final date of follow-up.

### Data collection

We obtained the demographic data, medical history, underlying comorbidities, clinical symptoms and signs, laboratory findings, chest computed tomography (CT), treatment measures, and outcome data from electronic medical records for all hospitalized patients with laboratory confirmed COVID-19. ARDS and shock were defined in accordance with the guidance of WHO for COVID-19 [10]. The diagnosis of acute kidney injury was based on the highest serum creatinine level and urine output [11]. Cardiac injury was defined if the serum concentration of hypersensitive cardiac troponin T (cTnT) was above the upper limit of the reference range (>0.02 ng/mL). Acute heart failure was defined based on the typical symptoms that may be accompanied by signs caused by a structural and/or functional cardiac abnormality [12]. Hypertension is defined in adults as the results of systolic blood pressure ≥ 140 mmHg and/or diastolic blood pressure ≥ 90 mmHg three times on different days. History of coronary heart disease was defined as having any of the following: evidence of myocardial infarction on the baseline electrocardiograph, self-report of a prior history of a cardiac procedure (coronary artery bypass surgery, coronary angioplasty, balloon angioplasty, atherectomy, stent, percutaneous transluminal coronary angioplasty, or percutaneous coronary intervention), self-reported history of myocardial infarction, or angina with ≥50% angiographic obstruction of a major coronary artery. The information was validated by hospital records.

### Statistical analysis

Continuous variables were presented as means and standard error (SEM) or medians and interquartile ranges (IQR) values as appropriate. Categorical variables were expressed as the counts and percentages in each category. Independent group t-test was used for continuous variable, when the data conformed to normal distribution; otherwise, the Mann-Whitney test was applied. Data (not normal distributed) from repeated measures were compared using the generalized linear mixed model. The chi-square test and Fisher’s exact test were applied for categorical variables as appropriate. Two-sided *p*-values less than 0.05 were considered statistically significant. All analyses were conducted with GraphPad Prism software (version 8.0).

## Results

### Baseline characteristics

The study population included 116 hospitalized patients with laboratory-confirmed COVID-19 (Table 1). The median age was 58.5 years (IQR, 47.0–69.0), and 36 (31.0%) were females. Fifty-nine (50.9%) patients had at least one underlying disorder. Hypertension (45 [38.8%]), diabetes (19 [16.4%]), coronary heart disease (17 [14.7%]), and cerebrovascular diseases (8 [6.9%]) were the most common coexisting conditions (Table 1). Fever (85.3%), dry cough (52.6%), fatigue (51.7%), anorexia (43.1%), dyspnea (44.8%), and chest discomfort (43.1%) were the most common symptoms, whereas dizziness (6.0%), nasal obstruction (5.2%), abdominal pain (2.6%), hemoptysis (0.9%) were less common (Table 1). The median durations from first symptoms to dyspnea and hospital admission were 4.5 days (IQR, 0–9.0), and 8.0 days (IQR, 4.0–11.0) respectively (Table 1).

**Table 1:** Baseline Characteristics of Patients Infected With COVID-19

	Disease severity			*P*Value
	Total (*N* = 116)	Non-severe (*n* = 61)	Severe (*n* = 55)	
**Age, median (IQR), y**	58.5 (47.0–69.0)	56.0 (37.0–64.0)	64.0 (53.0–76.0)	<0.001
0–14 y	0	0	0	–
15–49 y	33/116 (28.4)	24/61 (39.3)	9/55 (16.4)	–
50–64 y	41/116 (35.3)	23/61 (37.7)	18/55 (32.7)	–
≥ 65 y	42/116 (36.2)	14/61 (23.0)	28/55 (50.9)	–
**Sex –No., %**				
Female	36/116 (31.0)	19/61 (31.1)	17/55 (30.9)	1.000
Male	80/116 (69.0)	42/61 (68.9)	38/55 (69.1)	
**Comorbidities –No., %**				
Any	59/116 (50.9)	24/61 (39.3)	35/55 (63.6)	0.009
COPD	1/116 (0.9)	0/61 (0.0)	1/55 (1.8)	0.290
Diabetes	19/116 (16.4)	8/61 (13.1)	11/55 (20.0)	0.317
Hypertension	45/116 (38.8)	19/61 (31.1)	26/55 (47.3)	0.075
Coronary heart disease	17/116 (14.7)	4/61 (6.6)	13/55 (23.6)	0.009
Cerebrovascular disease	8/116 (6.9)	1/61 (1.6)	7/55 (12.7)	0.019
Malignancy	4/116 (3.4)	0/61 (0.0)	4/55 (7.3)	0.032
Chronic kidney disease	0/116 (0.0)	0/61 (0.0)	0/55 (0.0)	–
Chronic liver disease	2/116 (1.7)	1/61 (1.6)	1/55 (1.8)	0.941
HIV infection	0/116 (0.0)	0/61 (0.0)	0/55 (0.0)	–
**Signs and symptoms–No., %**				
Fever	99/116 (85.3)	49/61 (80.3)	50/55 (91.0)	0.108
Fatigue	60/116 (51.7)	26/61 (42.6)	34/55 (61.9)	0.039
Dry cough	61/116 (52.6)	31/61 (50.8)	30/55 (54.5)	0.688
Anorexia	50/116 (43.1)	27/61 (44.3)	23/55 (41.8)	0.791
Myalgia	32/116 (27.6)	19/61 (31.1)	13/55 (23.6)	0.366
Dyspnea	52/116 (44.8)	21/61 (34.4)	31/55 (56.4)	0.018
Expectoration	31/116 (26.7)	17/61 (27.9)	14/55 (25.5)	0.769
Hemoptysis	1/116 (0.9)	0/61 (0.0)	1/55 (1.8)	0.290
Pharyngalgia	15/116 (12.9)	7/61 (11.5)	8/55 (14.5)	0.623
Nasal obstruction	6/116 (5.2)	4/61 (6.6)	2/55 (3.6)	0.478
Diarrhea	17/116 (14.7)	14/61 (23.0)	3/55 (5.5)	0.008
Nausea	10/116 (8.6)	4/61 (6.6)	6/55 (11.0)	0.404
Dizziness	7/116 (6.0)	2/61 (3.3)	5/55 (9.1)	0.189
Headache	6/116 (5.2)	5/61 (8.2)	1/55 (1.8)	0.121
Vomiting	5/116 (4.3)	3/61 (4.9)	2/55 (3.6)	0.734
Chill	24/116 (20.7)	13/61 (21.3)	11/55 (20.0)	0.901
Shiver	5/116 (4.3)	3/61 (4.9)	2/55 (3.6)	0.734
Abdominal pain	3/116 (2.6)	3/61 (4.9)	0/55 (0.0)	0.096
Chest discomfort	50/116 (43.1)	22/61 (36.1)	28/55 (51.0)	0.107
Palpitation	13/116 (11.2)	7/61 (11.5)	6/55 (11.0)	0.923
Sleep disorders	27/116 (23.3)	15/61 (24.6)	12/55 (21.8)	0.724
**Onset of symptom to, median (IQR), d**				
Hospital admission	8.0 (4.0–11.0)	8.0 (4.0–11.0)	8.0 (4.0–11.0)	0.910
Dyspnea	4.5 (0.0–9.0)	3.0 (0.0–12.0)	6.0 (1.5–8.5)	0.649
Heart rate on admission, median (IQR), bpm	86.0 (80.0–98.0)	85.5 (80.0–97.5)	86.0 (80.0–98.0)	0.958
Respiratory rate on admission, median (IQR)	19.0 (18.0–23.0)	19.0 (18.0–20.0)	20.0 (18.0–25.0)	0.030
Mean arterial pressure on admission, median (IQR), mmHg	96.7 (86.7–103.3)	96.7 (86.7–103.5)	96.7 (86.7–103.3)	1.000
Temperature on admission, median (IQR), °C	36.8 (36.5–37.5)	36.7 (36.5–37.3)	37.0 (36.4–37.8)	0.138

On admission, 61 and 55 patients were categorized into non-severe and severe subgroups, respectively. The age differed significantly between the two groups (median age, non-severe vs severe, 56.0 years [IQR, 37.0–64.0] vs 64.0 years [IQR, 53.0–76.0]; *P* < 0.001). Severe cases were more prone to having underlying comorbidities, including coronary heart disease (13 [23.6%] vs 4 [6.6%], cerebrovascular diseases (7 [12.7%] vs 1 [1.6%]), and malignancy (4 [7.3%] vs 0 [0.0%]). Compared with the non- severe group, dyspnea and fatigue were more frequently reported in severe patients. Vital signs were recorded on the day of admission to hospital for all patients. Respiratory rate was higher in severe cases as compared with non-severe cases (20.0 [IQR, 18.0–25.0] vs 19.0 [IQR, 18.0–20.0; *P* = 0.030]). While, heart rate, mean arterial pressure, and body temperature showed no significant difference between the two groups (all *P*>0.05).

### Laboratory and radiologic findings at presentation

The most common pattern on chest CT was bilateral patchy shadowing (69.9%). These imaging alterations were more prominent in severe patients (47 [85.5%] vs 36 [59.0%]; *P* = 0.002) (Table 2).

**Table 2:** Chest Computed Tomographic Images Findings of Patients Infected With COVID-19 on Admission to Hospital

Abnormalities on chest CT –No./total No. (%)	Disease severity			*P*Value
	Total (*N* = 116)	Non-severe (*n* = 61)	Severe (*n* = 55)	
Local patchy shadowing	19/116 (16.4)	14/61 (23.0)	5/55 (9.1)	0.044
Bilateral patchy shadowing	81/116 (69.9)	36/61 (59.0)	47/55 (85.5)	0.002
Interstitial abnormalities	4/116 (3.4)	2/61 (3.3)	2/55 (3.6)	0.916

There were numerous differences in laboratory findings between severe and non-severe cases (Table 3). Laboratory abnormalities were more obviously seen in severe cases, including lower counts of lymphocyte, T cells, CD4^+^ and CD8^+^ T cells, and elevated levels of neutrophil count, procalcitonin, c-reactive protein, interleukin 6, D-dimer, creatinine, blood urea nitrogen, lactate dehydrogenase, myoglobin, cTnT, and NT-proBNP (all *P* < 0.05). Hypokalemia (20.7%) was prevailing in both severe and non-severe patients, though no statistical difference was found between them.

**Table 3:** Laboratory Findings of Patients Infected With COVID-19 on Admission to Hospital

			Disease severity		
Laboratory findings	Normal Range	Total (*N* = 116)	Non-severe (*n* = 61)	Severe (*n* = 55)	*P*Value
White blood cell count, ×109/L	3.5–9.5	4.9 (3.9–6.1)	5.0 (3.9–5.7)	4.9 (3.8–7.2)	0.3442
>9.5 × 109/L, No./total No. (%)		9/116 (7.8)	1/61 (1.6)	8/55 (14.5)	0.0129
<3.5 × 109/L, No./total No. (%)		17/116 (14.7)	9/61 (14.8)	8/55 (14.5)	1.0000
Neutrophil count, ×109/L	1.8–6.3	3.1 (2.2–4.5)	2.9 (2.1–3.8)	3.4 (2.5–5.8)	0.0134
Lymphocyte count, ×109/L	1.1–3.2	1.0 (0.7–1.3)	1.2 (0.9–1.6)	0.9 (0.6–1.1)	< 0.0001
<1.1 × 109/L, No./total No. (%)		66/116 (56.9)	24/61 (39.3)	42/55 (76.4)	< 0.0001
Total T cell count, × 106/L	955–2860	590.5 (361.0–941.8)	915.0 (481.0–1246.0)	448 (238.5–644.0)	< 0.0001
<955 × 106/L, No./total No. (%)		65/84 (77.4)	27/43 (62.8)	38/41 (92.7)	0.0014
CD4+ T cell count, × 106/L	550–1440	335.0 (214.8–493.3)	458.0 (275.0–607.0)	262.0 (156.5–335.0)	0.0002
<550 × 106/L, No./total No. (%)		66/84 (78.6)	29/43 (67.4)	37/41 (90.2)	0.0158
CD8+ T cell count, × 106/L	320–1250	217.0 (111.3–359.8)	285.0 (144.0–435.0)	157.0 (78.5–297.0)	0.0052
<320 × 106/L, No./total No. (%)		46/84 (54.8)	13/43 (30.2)	33/41 (80.5)	< 0.0001
Monocyte count, ×109/L	0.1–0.6	0.4 (0.3–0.6)	0.4 (0.4–0.6)	0.4 (0.2–0.6)	0.4886
Platelet count, ×109/L	125–350	177.0 (137.0–230.0)	179.0 (145.0–236.0)	161.5 (121.8–218.3)	0.1166
Haemoglobinlevel, g/L	130–175	131.0 (118.0–140.0)	129.0 (120.0–140.5)	131.0 (115.0–140.0)	0.7728
Prothrombin time, s	10.0–14.0	12.2 (11.5–13.0)	11.9 (11.5–13.0)	12.4 (11.6–13.1)	0.3330
Activated partial thromboplastin time, s	23.5–39.1	32.3 (30.1–34.8)	32.3 (30.2–34.6)	32.6 (29.8–35.0)	0.8014
D-dimer, ng/mL	0–243	168.0 (92.0–393.5)	122.0 (75.0–254.0)	222.0 (132.5–529.0)	0.0004
>243 ng/mL, No./total No. (%)			15/61 (24.6)	25/55 (45.5)	0.0204
Creatinine, umol/L	45–110	69.5 (56.0–81.0)	68.5 (54.3–75.8)	72.0(58.8–86.5)	0.0156
>110umol/L, No./total No. (%)		8/116 (6.9)	0/61 (0)	8/55 (14.5)	0.0019
Blood urea nitrogen, mmol/L	2.5–6.3	4.4 (3.4–5.8)	3.9 (3.2–5.0)	4.9 (3.5–7.0)	0.0080
>6.3 mmol/L, No./total No. (%)		22/116 (19.0)	5/61 (8.2)	17/55 (30.9)	0.0021
Alanine aminotransferase, U/L	9–50	24.0 (17.3–37.8)	22.0 (15.5–37.5)	27.0 (20.0–39.0)	0.1227
>50.0 U/L, No./total No. (%)		23/116 (19.8)	11/61 (18.0)	12/55 (21.8)	0.6470
Aspartate aminotransferase, U/L	15–40	34.5 (25.0–52.8)	31.0 (22.5–49.5)	38.0 (26.0–59.0)	0.0810
>40.0 U/L, No./total No. (%)		46/116 (39.7)	21/61 (34.2)	25/55 (45.5)	0.2573
Total bilirubin, umol/L	4.42–20.52	11.0 (8.1–14.1)	11.0 (8.3–13.0)	10.5 (7.8–16.3)	0.9308
Creatinekinase–MB, U/L	0–24	17.0 (15.0–21.0)	17.0 (15.0–22.5)	18.0 (15.8–20.0)	0.6960
>24.0 U/L, No./total No. (%)		19/116 (16.4)	12/61 (19.7)	7/55 (12.7)	0.4518
Lactate dehydrogenase, U/L	109–225	245.5 (194.3–319.8)	225.0 (184.0–303.5)	275.0 (212.0–378.0)	0.0041
>225.0 U/L, No./total No. (%)		69/116 (59.5)	30/61 (49.2)	39/55 (70.9)	0.0230
Hypersensitive troponin T, ng/mL	<0.02	0.008 (0.005–0.013)	0.007 (0.004–0.01)	0.0115 (0.006–0.0175)	0.0015
>0.02 ng/mL, No./total No. (%)		16/116 (13.8)	4/61 (6.6)	12/55 (21.8)	0.0288
Myoglobin, ng/mL	28–72	39.4 (22.3–92.7)	26.1 (21.0–47.8)	54.1 (33.4–120.7)	0.0002
>72.0 ng/mL, No./total No. (%)		34/116 (29.3)	12/61 (19.7)	22/55 (40.0)	0.0096
NT-proBNP, pg/mL		71.5 (27.0–363.5)	37.0 (19.0–118.8)	233.0 (53.3–693.0)	< 0.0001
elevated cases, No./total No. (%)		8/116 (6.9)	1/61 (1.6)	7/55 (12.8)	0.0260
Procalcitonin, ng/mL	0.00–0.50	0.07 (0.03–0.16)	0.05 (0.03–0.09)	0.09 (0.06–0.23)	< 0.0001
≥ 0.5 ng/mL, No./total No. (%)		8/116 (6.9)	2/61 (3.3)	6/55 (10.9)	0.1473
IL-6, pg/mL	0–7.0	18.7 (7.7–46.4)	11.8 (3.3–27.5)	33.0 (13.1–69.2)	< 0.0001
C-reactive protein level, mg/L	0–10	15.1 (9.0–50.9)	10.0 (7.0–27.5)	40.0 (10.0–8.0)	0.0023
Sodium, mmol/L	137.0–147.0	138.8 (137.1–141.0)	140.0 (138.2–141.7)	138.0 (134.8–139.3)	0.0001
Potassium, mmol/L	3.5–5.3	4.0 (3.5–4.3)	3.9 (3.5–4.3)	4.1 (3.6–4.3)	0.4548
>5.3 mmol/L, No./total No. (%)		1/116 (0.9)	0/61 (0)	1/55 (1.8)	0.4741
<3.5 mmol/L, No./total No. (%)		24/116 (20.7)	12/61 (19.7)	12/55 (21.8)	0.8213
Chloride, mmol/L	99.0–110.0	103.3 (99.8–105.5)	104.1 (101.3–106.2)	102.2 (99.2–103.8)	0.0011
Calcium, mmol/L	2.02–2.6	2.10 (2.04–2.21)	2.14 (2.09–2.28)	2.07 (2.02–2.13)	< 0.0001
Magnesium, mmol/L	0.6–1.2	0.73 (0.68–0.78)	0.76 (0.70–0.79)	0.70 (0.64–0.76)	0.0025

### Complications, main interventions, and outcomes

The organ dysfunction and treatment of the 116 patients were shown in Table 4. On April 21, 2020, a total of 109 patients (94.0%) had been discharged, and 7 patients (6.0%) had died. Among the 116 patients, the most common complication was acute cardiac injury (23 [19.8%]), followed by acute heart failure (21 [18.1%]), ARDS (20 [17.2%]), shock (16 [13.8%]), or liver dysfunction (15 [12.9%]). Severe cases were more liable to having one of these complications than non- severe cases.

**Table 4:** Complications, treatment and outcomes of patients with COVID-19

Characteristics	Total (*N* = 116)	Disease severity		*P*Value
		Non-severe (*n* = 61)	Severe (*n* = 55)	
**Complications, No./total No. (%)**				
Shock	16/116 (13.8)	0/61 (0.0)	16/55 (29.1)	< 0.0001
Acute cardiac injury	23/116 (19.8)	4/61 (6.6)	19/55 (34.5)	0.001
Acute respiratory distress syndrome	20/116 (17.2)	0/61 (0.0)	20/55 (36.4)	< 0.0001
Liver dysfunction	15/116 (12.9)	2/61 (3.3)	13/55 (23.6)	0.0011
Acute kidney injury	3/116 (2.6)	0/61 (0.0)	3/55 (5.5)	0.0565
Acute heart failure	21/116 (18.1)	3/61 (4.9)	18/55 (32.7)	0.0002
**Treatment, No./total No. (%)**				
Administration of oseltamivir	91/116 (78.4)	46/61 (75.4)	45/55 (81.8)	0.4019
Administration of lopinave/litonawe (LPV/r)	74/116 (63.8)	42/61 (68.9)	32/55 (58.2)	0.2325
Administration of arbidolhydrochloride	27/116 (23.3)	15/61 (24.6)	12/55 (21.8)	0.7243
Administration of ribavirin injection	71/116 (61.2)	37/61 (60.7)	34/55 (61.8)	0.8979
Administration of antibiotics	113/116 (97.4)	58/61 (95.1)	55/55 (100.0)	0.0956
Use of antifungal medications	12/116 (10.3)	0/61 (0.0)	12/55 (21.8)	0.0001
Administration of systemic corticosteroids	55/116 (47.4)	14/61 (23.0)	41/55 (74.5)	< 0.0001
Oxygen inhalation	103/116 (88.8)	50/61 (82.0)	53/55 (96.4)	0.0141
Noninvasive ventilation	18/116 (15.5)	0/61 (0.0)	18/55 (32.7)	< 0.0001
Invasive mechanical ventilation	10/116 (8.6)	0/61 (0.0)	10/55 (18.2)	0.0005
Extracorporeal membrane oxygenation	3/116 (2.6)	0/61 (0.0)	3/55 (5.5)	0.0646
Use of intravenous immunoglobin	74/116 (63.8)	25/61 (41.0)	49/55 (89.1)	< 0.0001
Use of thymopeptidesinjection	92/116 (79.3)	45/61 (73.8)	47/55 (85.5)	0.1209
Traditional Chinese medical herbal treatment	103/116 (88.8)	55/61 (90.2)	48/55 (87.3)	0.2955
**Clinical outcomes, No./total No. (%)**				
Discharge from hospital	109/116 (94.0)	61/61 (100.0)	48/55 (87.3)	0.0040
Death	7/116 (6.0)	0/61 (0.0)	7/55 (12.7)	0.0040

All patients were treated in isolation. 112 (96.6%) patients received antiviral treatment, including oseltamivir phosphatecas (91, [78.4%]), lopinave/litonawe (74, [63.8%]), arbidol hydrochloride (27, [21.4%]), and ribavirin Injection (71, [61.2%]). Antibacterial therapy was performed for most patients (113, [97.4%]). Twelve (10.3%) patients adopting antifungal medications were all enrolled in severe group. Systemic corticosteroid was given to 47.4% of cases and more so in the severe patients than in the non-severe group (74.5% vs 23.0%, *P* < 0.0001). Obviously, more severe cases received mechanical ventilation (non-invasive: 32.7% vs. 0%, *P* < 0.0001; invasive: 18.2% vs. 0%, *P* < 0.0001) as compared with non-severe cases. Three severe patients were treated with extracorporeal membrane oxygenation. Traditional Chinese medical herbal treatment and immune support therapy were applied in most cases. Overall, all these interventions were initiated in significantly higher percentages of severe patients.

Of the 7 deceased patients, all patients developed ARDS, 4 had sepsis or sepsis shock, 1 had gastrointestinal and cerebral hemorrhage. As the disease progressed and clinical status deteriorated, the levels of D-dimer, creatinine, blood urea, lactate dehydrogenase, and cTnT progressively increased before death. Among the dead patients, all received antiviral therapy, antibacterial therapy, and immune support therapy (use of intravenous immunoglobin and/or thymopeptides injection). Based on the 6th edition guideline issued by the National Health Commission of China, Hydroxychloroquine had not been recommend in the treatment of COVID-19 yet. Meanwhile, traditional Chinese medical herbal treatment, including Lianhua Qingwen Capsule and oral use of Chinese herbal decoction, like Lung cleansing and detoxification soup, were recommended and applied in most hospitalized patients. This part of treatment was managed by the doctor majoring in traditional Chinese medicine. In the present study, 5 non-survivors received Lianhua Qingwen Capsule therapy.

### Dynamic profile of blood pressure and cardiac markers

**Fig. 1:**
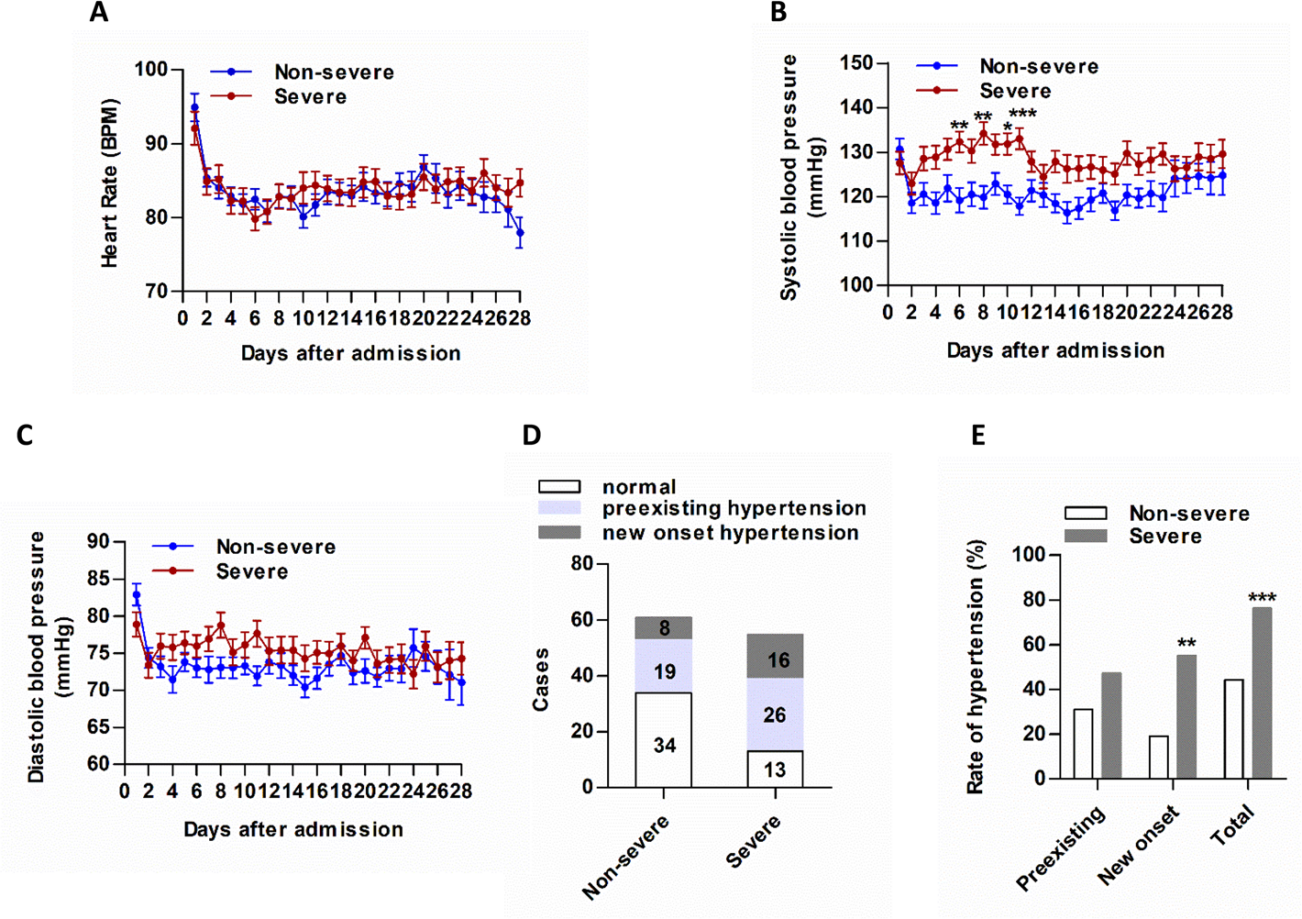
Dynamic monitoring heart rate and blood pressure of patients hospitalized with COVID-19. During hospitalization, heart rate and blood pressure were recorded every day at the same time by nurses. The dynamic monitoring results of heart rate (**a**), systolic blood pressure (**b**), and diastolic blood pressure (**c**) were compared between non-severe and severe cases. **d** The counts of normal blood pressure, preexisting hypertension, and new onset hypertension cases in non-severe and severe groups. **e** Rate of preexisting hypertension, new onset hypertension, and the total hypertension were compared between non-severe and severe subgroups. *P < 0.05, **P < 0.01, ***P < 0.001 for Non-severe vs Severe

**Fig. 2:**
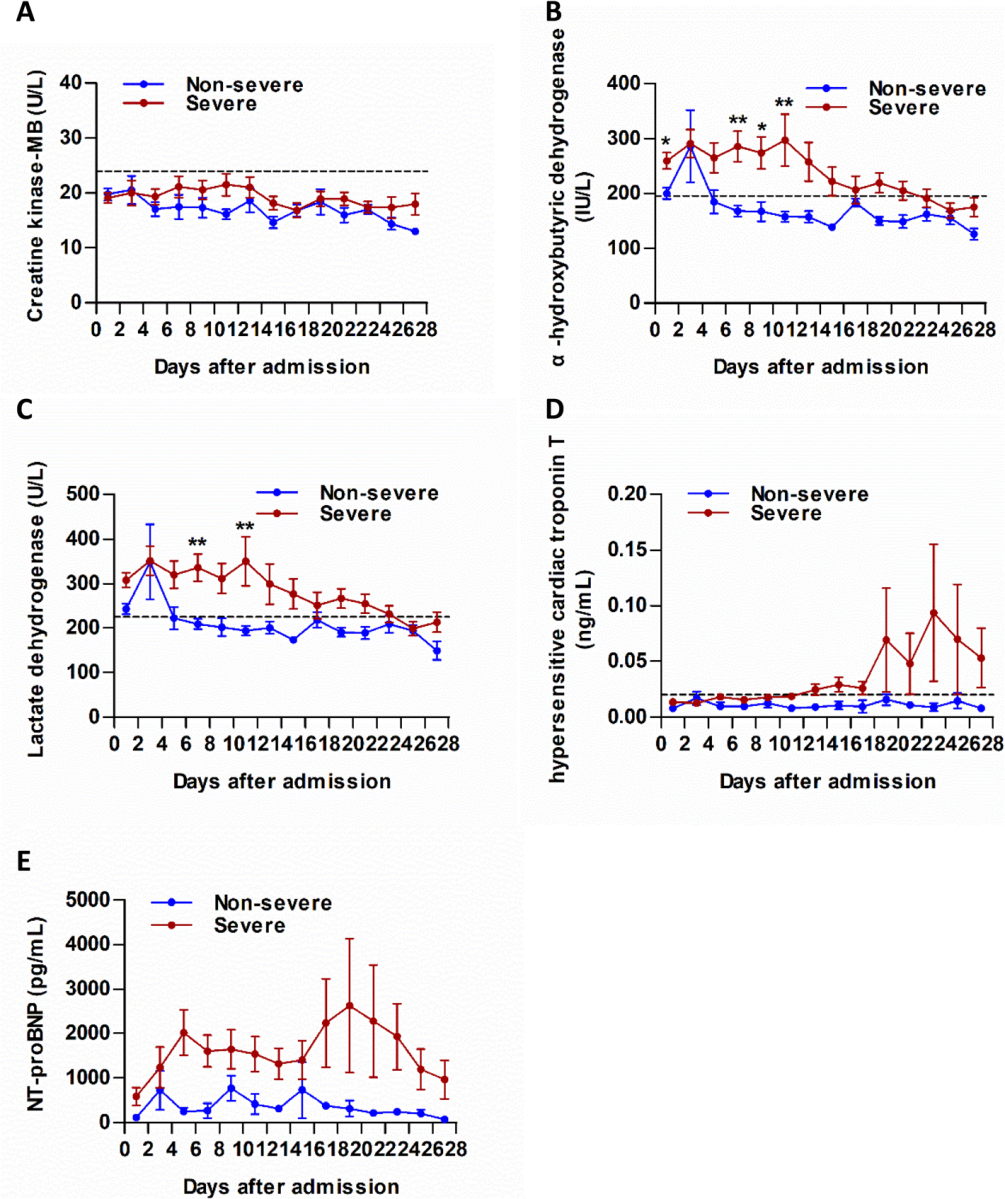
Temporal changes in laboratory markers in patients hospitalized with COVID-19. Figure shows temporal changes in creatine kinase-MB (**a**), α-hydroxybutyric dehydrogenase (**b**), lactate dehydrogenase (**c**), cTnT (**d**), and NT-proBNP (**e**) of non-severe and severe patients every other day after admission. The dotted lines in black show the lower limit of normal for each parameter. *P < 0.05, **P < 0.01 for Non-severe vs Severe

To determine the cardiovascular implications during COVID-19 progression, the dynamic changes in heart rate, blood pressure, and 5 clinical laboratory parameters, including creatine kinase-MB, α-hydroxybutyric dehydrogenase, lactate dehydrogenase, cTnT, and NT-proBNP were tracked in survivors (Figs. 1 and 2). The results of heart rate and blood pressure were recorded every day at the same time. Laboratory parameters were examined once every 3 days. During hospitalization, higher levels of systolic blood pressure were observed in server cases (Fig. 1b). A total of 8 patients from non-severe group and 16 patients from severe group were diagnosed with new onset hypertension (Fig. 1d). The morbidity rate of hypertension was significantly higher in severe cases (42 [76.4%] vs 27 [44.3%]; *P* = 0.0006) (Fig. 1e).

During hospitalization, severe cases exhibited higher levels of cardiac markers (Fig. 2). The prevalence of cardiac complications, including acute cardiac injury and heart failure was significantly higher in severe cases (Table 4). Increases of lactate dehydrogenase and α-hydroxybutyric dehydrogenase were also more likely to occur in severe patients (Fig. 2b, c). Myocardial enzymes increased rapidly in the early stage of illness, but gradually decreased with the disease progression. In the end period of the 28-day time frame, abnormal results of cTnT and NT-proBNP were still common in some of the severe patients because of progression of COVID-19 (Fig. 2d, e).

## Discussion

This retrospective study described the clinical characteristics and cardiovascular implications in hospitalized patients with COVID-19 in Wuhan. By April 13, 2020, of the 116 patients included in this study, 69% were male, 47% were severe cases, 90.5% were discharged, 6.0% (7) died, and 3.4% remained hospitalized. Most severe patients were older and had more underlying conditions. Common symptoms at onset of illness were fever, dry cough, fatigue, dyspnea, and chest discomfort. Local and/or bilateral patchy shadowing was a typical hallmark of CT imaging for COVID-19. Lymphopenia and elevated levels of neutrophil count, C-reactive protein, interleukin 6, D-dimer, creatinine, lactate dehydrogenase, cTnT, and NT-proBNP were more commonly seen in severe cases. During hospitalization, the prevalence of new onset hypertension, acute heart injury, and heart failure was significantly higher in severe patients.

In our cohort, 69% (80) of COVID-19 patient were male. Severe patients were older and had a greater number of comorbid conditions. Evidence from previous studies suggest that older, male patients are the most susceptible to SARS-CoV-2 infection [4, 5, 7, 13], which is supported by our data. It has been confirmed that increased age was associated with death in COVID-19 patients [14], and the coexistence of agedness and comorbidity could lead to an even higher risk of death [13]. Older age has been regarded as an important independent predictor of mortality in COVID-19.

Cardiovascular diseases have a high incidence rate in the middle aged and elderly population [8]. As previously reported [14], we observed that many COVID-19 patients had a comorbidity, with hypertension being the most common (45 [38.8%]), followed by diabetes (19 [16.4%]) or coronary heart disease (17 [14.7%]). The morbidity rates of coronary heart disease and cerebrovascular diseases were significantly higher in the severe group. Thus, older people with comorbidities, such as coronary heart disease and hypertension were thought to be more vulnerable to SARS-CoV-2 and result in more severe outcomes and elevated case-fatality rate [5, 9, 15]. In the present study, 4 of 7 dead patients had preexisting hypertension and coronary heart disease. Previously, coronary heart disease has also been found to be correlated with acute cardiac events and poor outcomes in influenza and other respiratory viral infections [16, 17], Multivariate logistic regression analysis demonstrated that coronary heart disease and heart injury were the independent risk factors for critical disease status in COVID-19 patients [18]. More intense clinical care is in need for COVID-19 patients with cardiac-related chronic diseases.

Incident cardiovascular complications including new or worsening heart failure, new or worsening arrhythmias, or myocardial infarction are common in patients with pneumonia and are associated with increased short-term mortality [19]. Acute pneumonia brings important effects on the status of cardiovascular system irrespective of severities of infection [16, 19]. Risk factors for cardiac complications after pneumonia include older age, preexisting cardiovascular diseases, and greater severity of pneumonia [16, 19]. An analysis of 112 cardiovascular disease patients with COVID-19 found that, COVID-19 patients combined with cardiovascular disease were associated with a higher risk of mortality [15]. In this study, compared with non-severe patients with COVID-19, severe patients showed abnormalities in numerous cardiac markers. During hospitalization, the morbidity of new onset hypertension, acute heart injury, and heart failure was significantly higher in severe patients. Increased level of myocardial enzymes and cTnT was found in all 3 dead cases. As far as we know, this is the first study that reports the prevalence rate of new onset hypertension was significantly higher in hospitalized severe patients with COVID-19. These findings suggest a higher possibility of cardiovascular complications in severe patients with COVID-19. Outcomes of patients with COVID-19 may be improved by prevention of the development and progression of associated cardiac complications.

Angiotensin-converting enzyme 2 (ACE2) acts as a receptor for SARS-CoV-2 entry into cells and contributes to the pathogenesis of COVID-19 [20]. Meanwhile, ACE2 is widely expressed in myocytes and vascular endothelial cells. At least, these is theoretically a possibility of direct cardiovascular involvement induced by the virus. The result of heart biopsy in a fatal case with COVID-19 showed a few interstitial mononuclear inflammatory infiltration, but no other substantial damage in the heart tissue [21]. However, given that this patient had no clinical manifestations of myocardial injury during the whole course of this disease, it could not be concluded whether myocardium was involved in SARS-CoV-2 infection yet. The latest results of autopsy of COVID-19 victims in China demonstrated the existence of RNA and viral particles of SARS-CoV-2 in heart through qRT-PCR-based virus nuclear acid detection, electron microscopy, and immunohistochemical staining [22]. Myocardia displayed cell degeneration, scattered necrosis, interstitial edema, and mild infiltration of monocytes, lymphocytes, and/or neutrophils. Multiple postmortem regions showed tunica intima inflammation, thrombosis, anemic infarct [22]. We speculated that the potential pathogenesis of myocardial injury in COVID-19 may include several processes, SARS-CoV-2 may directly invade myocytes via ACE2 and cause viral myocarditis; the imbalance between supply and demand in oxygen further results in myocardial injury; and inflammatory cytokines storm. In order to further clarify the etiology of SARS-CoV-2 infection related myocardial injury, it is of great need to obtain pathological evidence from COVID-19 patients showing definite myocardial injury.

Recently, the safety of treatment applying angiotensin converting enzyme inhibitors (ACEI) or angiotensin receptor blockers (ARBs) in relation to COVID-19 has been concerned. An observational study containing 112 patients with cardiovascular diseases infected by COVID-19 reported that there was no significant difference in the proportion of ACEI/ARB medication between the critical group and the general group or between non-survivors and survivors [15]. Currently, it is in lack of any experimental or clinical evidence suggesting adverse or beneficial outcomes with background use of ACEI, ARBs or other RAAS antagonists in COVID-19 or among COVID-19 patients with a history of cardiovascular disease treated with such agents. Statements of ACC and ESC Council on Hypertension do not recommend to discontinue ACEI/ARB treatment in the lack of any evidence supporting adverse effect of ACEI and ARB in the context of the pandemic COVID-19 outbreak [23, 24]. Individualized treatment strategies should be approached according to each patient’s hemodynamic status and clinical manifestations.

Our study has some limitations. First, only 116 patients with laboratory-confirmed COVID-19 were included. It would be better to include a large population of patients from other centers in Wuhan, and even in other cities in China to get a more comprehensive understanding of COVID-19. Second, not all laboratory tests were dynamically performed in all patients, including the counts of lymphocyte subsets and inflammatory cytokines, therefore their role in the pathogenesis of COVID-19 might be underestimated. Third, due to the retrospective study design, echocardiography and electrocardiograph were only performed in some of the patients. The detailed information of ACEI/ARB medication was incomplete. Therefore, we could not further asses the changes of cardiac structure and function during the progression of COVID-19, and the possible effect of ACEI/ARB on SARS-CoV-2 infection. Last but not least, we were unable to obtain myocardial tissues from deceased COVID-19 patients with heart injury. The characteristics of myocardial damage should be further demonstrated by pathologists.

## Conclusions

In this single-centered, retrospective, observational study, we found that the infection of SARS-CoV-2 were more likely to occur in older population with cardiovascular comorbidities. Cardiovascular complications, including new onset hypertension and heart injury were common in severe patients with COVID-19. More comprehensive and in-depth researches are in need to unveil the cardiovascular involvement in COVID-19 to further understand the disease.

## Data Availability

The datasets used and/or analyzed during the current study are available from the corresponding author on reasonable request.

## Abbreviations

COVID-19: Corona Virus Disease 2019
SARS-CoV-2: 2019 novel coronavirus
ARDS: Acute respiratory distress syndrome
cTnT: Hypersensitive cardiac troponin T
NT-proBNP: N-terminal B-type natriuretic peptide
ACC: American College of Cardiology
ESC: European Society of Cardiology
SEM: Standard error
IQR: Interquartile ranges
